# *Lars2* Deficiency-Induced Mitochondrial Dysfunction Drives the Emergence of a Pro-Inflammatory Stroke-Specific Microglial Subpopulation

**DOI:** 10.14336/AD.2025.0387

**Published:** 2026-06-16

**Authors:** Qing Zou, Jianxin Zhou, Ying Li, Jiaming Shi, Jingying Huang, Cheng Zhuang, Hao Wu, Huanle Hong, Yanan Guo, Qian Li, Robert Chunhua Zhao, Jiao Wang

**Affiliations:** ^1^School of Life Sciences, Shanghai University, Shanghai, China.; ^2^Institute of Basic Medical Sciences Chinese Academy of Medical Sciences, School of Basic Medicine Peking Union Medical College, Beijing, China.; ^3^Centre of Excellence in Tissue Engineering, Chinese Academy of Medical Sciences, Beijing, China.; ^4^Beijing Key Laboratory of New Drug Development and Clinical Trial of Stem Cell Therapy (BZ0381), Beijing, China.; ^5^Cell Energy Life Sciences Group Co. LTD, Qingdao, China.; ^6^School of Life Science and Technology, Shandong Vocational University of Foreign Affairs, Jinan, China.

**Keywords:** Stroke, neuroinflammation, microglial subpopulation, mitochondria, *Lars2*

## Abstract

Stroke significantly alters microglial immune status beyond the traditional M1/M2 classification. We analyzed single-cell RNA sequencing data from the striatum of hemorrhagic, ischemic, and control mice, revealing activation of mitochondrial autophagy and assembly processes after stroke. Gene Ontology functional enrichment analysis indicated that stroke-associated genes predominantly regulate mitochondrial maintenance, with leucyl-tRNA synthetase 2 (*Lars2*) markedly upregulated in post-stroke microglia. A distinct microglial subset (Mc) was identified with notably low *Lars2* expression. *In vitro*, *Lars2* overexpression enhanced mitochondrial function, reduced pro-inflammatory cytokine release, and suppressed Mc marker gene expression. Cell-cell communication analysis revealed Mc as the most interactive microglial subset following stroke, particularly engaging with neurons. Among neuron-Mc signaling pairs, the neurotrophic factor pleiotrophin-syndecan-4 (PTN-SDC4) ligand-receptor pair emerged as a key mediator. Conditioned media from stressed microglia upregulated neuronal *Ptn* expression, likely recruiting microglia, as exogenous PTN promoted microglial migration. These findings identify Mc as a stroke-induced microglial population with low *Lars2* expression and pro-inflammatory features. The lack of compensatory mitochondrial repair in Mc contributes to pro-inflammatory polarization, positioning *Lars2* as a mitochondrial checkpoint linking stroke-induced microglial reprogramming to neuroinflammation.

## INTRODUCTION

Stroke is a leading cause of global mortality and long-term disability, with hemorrhagic and ischemic subtypes collectively imposing substantial socioeconomic burdens [1]. While current neurorestorative strategies demonstrate modest efficacy in functional recovery, the role of microglia, the brain's resident immune sentinels, remains incompletely understood in post-stroke neuro-inflammation [2-5]. Historically, microglia have been dichotomized into pro-inflammatory M1 and anti-inflammatory M2 phenotypes; however, this binary framework inadequately reflects their complex immunological landscape [6]. To elucidate the full dynamics of microglia, sophisticated techniques such as transcriptomic or proteomic analyses on specific disease models are essential.

Here, we combined single-cell transcriptomics and functional assays in mouse models of ischemic and hemorrhagic stroke to define the role of microglial populations in driving pro-inflammatory events within the striatum [7]. Intriguingly, mitochondrial gene scoring revealed stroke-induced activation of mitochondrial autophagy and assembly pathways, particularly in newly recruited microglia, suggesting an adaptive response to hypoxic-ischemic stress. We further identified a stroke-specific microglial subpopulation, designated Mc, characterized by pro-inflammatory signatures (*Cst7,Apoe*) and impaired mitochondrial repair capacity. Central to this phenotype was the dysregulation of leucyl-tRNA synthetase 2 (*Lars2*), a critical regulator of mitochondrial integrity [8-10]. Prior studies have demonstrated that abnormalities in *Lars2* disrupt mitochondrial architecture and elevate mitochondrial reactive oxygen species (mtROS) [8]. While global *Lars2* upregulation post-stroke could suggest a compensatory mechanism, Mc uniquely exhibited suppressed *Lars2* expression, correlating with sustained mtROS accumulation, DRP1-mediated fission, and TNF-α/IL-6/IL-1β hypersecretion. Accordingly, *Lars2* overexpression restored mitochondrial membrane potential, attenuated mtROS levels, downregulated *Drp1* expression, inhibited the pro-inflammatory polarization of Mc microglia, and reinstated the expression of homeostatic marker *P2ry12*. Conversely, knockdown of *Lars2* exacerbated inflammatory cascades. In summary, *Lars2* upregulation post-stroke supports mitochondrial recovery and mitigates microglial inflammatory responses. Its deficiency promotes the emergence of a stroke-specific mitochondrial-associated Mc microglial subset with pro-inflammatory properties. We propose that stroke-induced mitochondrial dysfunction drives microglial polarization through *Lars2* dysregulation, highlighting this axis as a potential therapeutic target to alleviate post-stroke neuroinflammation.

## MATERIALS AND METHODS

### Animals

Male C57BL/6 wild-type (WT) mice, weighing 22-25 g and aged 8-10 weeks, were used in this study. Animals were housed under standardized conditions (22 ± 1°C, 60-80% humidity, 12/12 h light/dark cycle) with food and water *ad libitum* in a specific pathogen-free (SPF) animal facility at Shanghai University. All experimental procedures were approved by the Shanghai University Animal Experimentation Committee.

### Establishment of the stroke models

Ischemic stroke (IS) was induced via transient middle cerebral artery occlusion (MCAO) following established protocols. Briefly, mice were anesthetized with 1% chloral hydrate. After sterilization, a cervical incision was made to expose the external carotid artery, through which a filament was advanced into the internal carotid artery to occlude the origin of the middle cerebral artery. Reperfusion was performed by withdrawing the filament 90 min after occlusion.

For the hemorrhagic stroke (HS) model, mice were anesthetized with 1% chloral hydrate. A total of 0.075 U collagenase IV dissolved in 4 μl PBS was stereotactically injected into the brain at specified coordinates (2.2 mm left, 1.0 mm anterior, and 2.7 mm deep, relative to the anterior fontanelle). The injection needle was retained in place for 10 min post-injection to prevent back flow. The body temperature was maintained at 37°C throughout the procedure.

### RNA interference

To knock down *Lars2* or *E2f1* in BV2 microglial cells, three siRNAs were synthesized for each gene (GeneAdv, Suzhou, China): *Lars2*-siRNA-1,2,3; *E2f1*-siRNA-1, 2, 3 (Table 1). Transfections were performed using the EZ Trans siRNA reagent (Life-iLab). Cells were incubated in Opti-MEM (Invitrogen) containing 20 nM *Lars2/E2f1*-siRNA and Lipofectamine for 4 h. Subsequently, the transfection medium was replaced with fresh culture medium, and cells were incubated for an additional 24 h during which they were subjected to oxygen-glucose deprivation/reperfusion (OGD/R) or lipopolysaccharide (LPS) treatment at specified time points. Through qPCR analysis, *Lars2*-siRNA-1 and *E2f1*-siRNA-3 were found to exhibit the highest silencing efficiency, which were used for subsequent experiments (Supplementary Fig. 1).

### Plasmid construction and transfection

To overexpress *Lars2* in BV2 cells, the *Lars2* sequence (NCBI RefSeq: NM-102436) was synthesized with mammalian codon optimization and cloned into the pcDNA3.1-T2A vector (GeneAdv, Suzhou, China) between EcoRI and XhoI sites. The construct contained an N-terminal HIS tag for purification and a C-terminal EGFP2 tag for real-time quantification. The sequence was validated by Sanger sequencing. When cells reached approximately 70% confluency, plasmids were transfected using the EZ Trans lipo pro reagent (Life-iLab) according to the manufacturer’s instructions.

**Table 1:** Sequences of *Lars2- or E2f1*-siRNAs.

Gene	Forward (5’-3’)	Reverse (5’-3’)
**Negative control**	UUCUCCGAACGUGUCACGUTT	ACGUGACACGUUCGGAGAATT
***Lars2-*siRNA-1**	GGUGCAAGGGAUCAGCAAATT	UUUGCUGAUCCCUUGCACCTT
***Lars2-*siRNA-2**	GGUGGACACGUGACAAGUATT	UACUUGUCACGUGUCCACCTT
***Lars2-*siRNA-3**	GGAAGGCUCUCUAGAUUCATT	UGAAUCUAGAGAGCCUUCCTT
***E2f1-*siRNA-1**	CGCUAUGAAACCUCACUAATT	UUAGUGAGGUUUCAUAGCGTT
***E2f1-*siRNA-2**	CCAAGAAGUCCAAGAAUCATT	UGAUUCUUGGACUUCUUGGTT
***E2f1-*siRNA-3**	GCUGUGGAUUCUUCAGAGATT	UCUCUGAAGAAUCCACAGCTT

### Real-time quantitative PCR (RT-qPCR)

Total RNA was extracted from the hippocampal tissue using the RNA Isolater Total RNA Extraction Reagent (Vazyme, Nanjing, China) according to the manufacturer's protocol. The extracted RNA was reverse-transcribed into complementary DNA (cDNA). RT-qPCR was performed using SYBR Green Master Mix (Yeasen, Shanghai, China) with the following programme: 95°C for 1 min; 40 cycles of 95°C for 20 s; 60 repeats of an increment of 0.5°C from 65°C to 95°C, 5s for each repeat. The primers used for amplification were summarized in Table 2.

**Table 2:** Primer sequences for qPCR.

Gene	Forward (5’-3’)	Reverse (5’-3’)
***Lars2***	CATAGAGAGGAATTTGCACCCT	GCCAGTCCTGCTTCATAGAGTTT
***E2f1***	CTCGACTCCTCGCAGATCG	GATCCAGCCTCCGTTTCACC
***Ptn***	ATGTCGTCCCAGCAATATCAGC	CCAAGATGAAAATCAATGCCAGG
**Chip-qpcr-*Mfn2***	ACAGCAGAGGCAGAATGGAC	AGAGACACACCCAGCACTTG
**β-tubulin**	CGTTGACATCCGTAAAGACC	AACACGTCCGCCTAGAAGCAC

### Chromatin immunoprecipitation (ChIP)-qPCR

ChIP was performed using the Pierce Magnetic ChIP Kit 491 (Thermo Fisher Scientific, Boston, MA, USA) according to the manufacturer's instructions. In brief, cells were cross-linked with 1% formaldehyde for 10 min at room temperature, followed by the addition of 2.5 M glycine (a final concentration of 125 mM) for 5 min. The chromatin was sonicated to generate DNA fragments of 200-500 bp. Antibodies against E2F1 (HUABIO, ET1701-73) or IgG (Servicebio, GB111738-100) were added to precipitate the protein-DNA complexes. The chromatin DNAs were extracted using a DNA Purification Kit (Thermo Fisher Scientific, Boston, MA, USA) and were subjected to qPCR analysis with specific primers. The target sequence of the *Mfn2* promoter is shown in Supplementary Figure 2.

**Table 3:** Primary antibodies used for western blotting.

Antibody	Supplier / Catalog No.	Working concentration
**Anti-P2RY12**	Affinity/DF10263	1:1000
**Anti-LARS2**	Affinity/DF13122	1:1000
**Anti-CST7**	Abclonal/A8164	1:1000
**Anti-IL-6**	Proteintech/66146-1-Ig	1:1000
**Anti-IL-1β**	Proteintech/66737-1-Ig	1:1000
**Anti-TNFα**	Abclonal/A0277	1:1000
**Anti-DRP1**	Abclonal/A16661	1:1000
**Anti-PTN**	Santa Cruz/sc-74443	1:1000
**Anti-SDC4**	Proteintech/11820-1-AP	1:1000
**Anti-E2F1**	HUABIO/ET1701-73	1:1000
**Anti-MFN2**	Abmart/T56638	1:1000
**Anti-β-tubulin**	Abclonal/AC021	1:5000

### Western blotting

To extract total proteins, cells were collected and incubated in a lysis buffer (Beyotime, China), supplemented with phenylmethylsulfonyl fluoride (PMSF) and a phosphatase inhibitor cocktail (Solarbio, Beijing, China), following the manufacturer’s protocol. Samples from three independent experimental replicates were collected. Cellular homogenates were centrifuged at 12,000 rpm and 4°C for 30 min, and the supernatants were collected. Protein concentrations were determined using a BCA-100 protein assay kit (Beyotime, China).

The proteins (20 μg) were denatured by boiling in 5× SDS-PAGE loading buffer for 10 min and separated by SDS-PAGE on a 12% polyacrylamide gel. Proteins were transferred onto a nitrocellulose membrane, blocked with 5% bovine serum albumin (Solarbio, Beijing, China) for 2 h at room temperature, and incubated with primary antibodies (Table 3) overnight at 4°C, followed by incubation with horseradish peroxidase-conjugated secondary antibodies for 1 h at room temperature. Immunoreactive bands were visualized and quantified using an Odyssey scanner (LI-COR Biosciences, USA) and ImageJ, respectively. Relative protein expression levels were normalized to β-tubulin.

### Immunohistochemistry

Brain sections were equilibrated to room temperature and dried at 37°C for 10 min to increase tissue adherence to slides. Sections were washed three times in PBS for 5 min each, followed by permeabilization in PBS containing 0.6% Triton X-100 for 30 min. To minimize nonspecific binding, sections were blocked with 5% fetal bovine serum (FBS) in PBS for 2 h at room temperature. Then sections were incubated in primary antibodies diluted in the blocking buffer overnight at 4°C, washed three times with PBS for 10 min each, and incubated in secondary antibodies conjugated with fluorophores at 37°C for 2 h. After the incubation, sections were washed with PBS and stained with DAPI for 20 min at room temperature, followed by another three washes with PBS. Finally, sections were mounted with an anti-fade mounting medium (Life-iLab,AC38L532) and sealed with coverslips. Immunofluorescent images were captured using a fluorescence microscope (Carl Zeiss, Germany). IgG isotype and secondary-only controls served as negative controls (Supplementary Fig.3). The antibodies used for immunohistochemistry were listed in Table 4.

**Table 4:** Antibodies used for immunocytochemistry.

Antibody	Supplier / Catalog No.	Working concentration
**Anti-LARS2**	Affinity/DF13122	1:50
**Anti-CST7**	Abclonal/A8164	1:50
**anti-PTN**	Santa Cruz/sc-74443	1:50
**Anti-IBA1**	Abcam/Ab283319	1:100
**Anti-NEUN**	Yeasen Biotechnology/31082ES50	1:50
**Anti-Rabbit IgG**	Servicebio/GB111738-100	1:100
**Anti-Mouse IgG**	Servicebio/GB111739-100	1:100
**Goat Anti-Rabbit IgG H&L (Alexa Fluor 488)**	Abcam/ab150077	1:200
**Goat Anti-Mouse IgG H&L (Alexa Fluor 488)**	Abcam/ab150117	1:200
**Goat Anti-Rabbit IgG H&L (Alexa Fluor 594)**	Abcam/ab150080	1:200
**Goat Anti-Mouse IgG H&L (Alexa Fluor 594)**	Abcam/ab150116	1:200
**DAPI**	Servicebio/G1012-10ML	/

### Determination of mitochondrial membrane potential

The mitochondrial membrane potential was detected using a mitochondrial membrane potential assay kit with JC-1 (Beyotime, C2006). Briefly, an appropriate amount of JC-1 staining working solution was added to the plate wells, and incubated with cells at 37°C for 20 min. After the incubation, the culture medium and staining solution were aspirated, and the cells were washed twice with the staining buffer. Subsequently, new culture medium was added, and the cells were observed under a fluorescence microscope (Carl Zeiss, Germany). When mitochondrial membrane potential is high, JC-1 is driven into the mitochondrial matrix and forms red aggregates. When mitochondrial membrane potential is low, JC-1 retains in the cytosol as monomers and emits green fluorescence. The intensity ratio of red-to-green fluorescence was calculated to indicate the mitochondrial membrane potential.

### Measurement of mtROS

The intracellular mtROS level was quantified using MitoSOX™ Red (Invitrogen, M36009) according to the manufacture’s instructions. Briefly, cells were incubated with the working solution of MitoSOX™ Red (500 nM) for 30 min at 37°C, washed with PBS, and imaged using a fluorescence microscopy. The intensity of red fluorescence was measured as an indicator of mtROS levels, reflecting the levels of oxidative stress.

### scRNA-seq analysis

#### Data processing

The Seurat R package (version 4.0)[11] and the Scrublet software (version 0.2.2) [12] were used for the quality control and doublet removal, respectively. Then data from all samples were normalized and merged into a unified dataset. Transcript levels were normalized using Seurat's NormalizeData function, multiplied by a scale factor of 10,000 and log-transformed for data visualization and downstream analysis.

#### Identification of differentially-expressed genes (DEGs)

DEGs were identified using the Find Markers function in Seurat. The Model-based Analysis of Single-cell Transcriptomics (MAST) test was adopted to determine the statistical significance, applying thresholds of P value < 0.05 and |log2foldchange| > 0.58.

#### Dimension reduction and unsupervised clustering

Normalized data were analyzed to identify genes with high cell-to-cell variations by employing the FindVariableFeatures function. The top 2,000 highly variable genes were selected for scaling with the ScaleData function. Principal component analysis (PCA) was performed to reduce dimensionality. Next, graph-based clustering was applied using the FindNeighbors and FindClusters functions, with resolution optimized for distinct cluster separation. DEG markers in each cluster were identified with the FindAllMarkers function, which compares gene expression levels with those in all other cell clusters.

#### Cell type annotation

Cell types were annotated using the SingleR package (version 1.4.1), with reference to transcriptomic datasets of pure cell types[13]. Cluster annotations were validated against manually curated gene markers from the CellMarker database [14]. DEGs in each cell type were identified using the following thresholds: expression in at least 20% of cells in either sample; |log2FoldChange| > 0.585; adjusted p value < 0.01.

#### Functional enrichment analysis

Functional enrichment analysis was conducted using the ClusterProfiler R package (version 4.1) [15]. DEGs in each cell type or cluster were subjected to analyses of Gene Ontology (GO) or Kyoto Encyclopedia of Genes and Genomes (KEGG). Gene Set Enrichment Analysis (GSEA) was performed using the gsea function. GO terms or KEGG pathways with an adjusted p value < 0.05 were considered significantly enriched.

#### Cell-cell interaction network analysis

The R package CellChat (version 1.1.3) was used to identify the potential interactions between different cell populations [16]. The CellChatDB murine database was referred for ligand-receptor pairs and secreted signaling molecules for cell-cell communication.

#### Gene set activity scoring

The UCell R package (version 2.7.2) was used to estimate the activity of gene sets [17].

#### Statistics and reproducibility

Statistical analysis and data visualization were performed using the R software (version 4.0.2). Unless otherwise specified, statistical significance was defined as p < 0.05 or FDR < 0.05.

#### Pseudotime analysis and trajectory inference

Pseudotime trajectories were constructed using the differential GeneTest function to identify ordering genes that inform cell progression, simulating dynamic alterations of cellular state transitions during developmental processes e[18, 19].

#### Statistical analysis

All data were analyzed and visualized with GraphPad Prism 9.0.6. Quantitative results are presented as mean ± SEM in the figures. Normality was confirmed for all datasets by the Shapiro-Wilk test. Group comparisons were performed using One-way ANOVA with Tukey's *post hoc* test for three or more groups and paired or independent two-tailed Student’s t-test for two groups. P-values are reported in the figures.

## RESULTS

### Single-cell transcriptomic landscape of the striatum in control, HS, and IS mice

**Figure 1.**
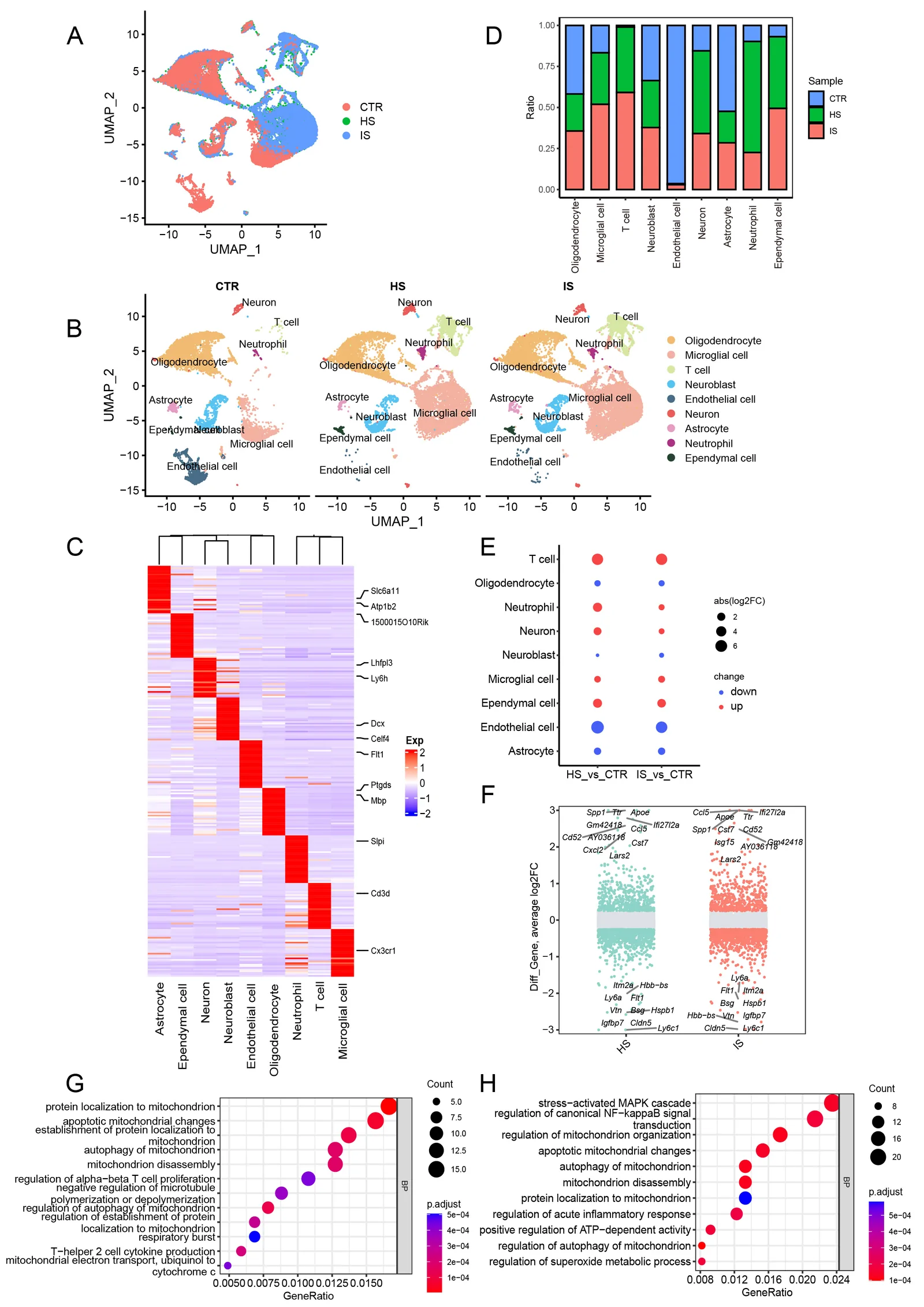
**Transcriptomic profiling of the striatum from control (CTR), hemorrhagic stroke (HS), and ischemic stroke (IS) mice**. (**A-B**) UMAP plots showing cell clusters in the striatum of CTR, HS, and IS mice, respectively. (**C**) Heatmap displaying the relative expression levels of top marker genes for each cell type. (**D**) Bar graph showing the proportions of cell clusters as identified in (B). (**E**) Dot plot illustrating changes in the proportions of different cell types in the striatum of HS or IS mice compared with CTR. Red and blue denote upregulation and downregulation, respectively. (**F**) Volcano plot demonstrating DEGs in the striatum of HS or IS mice compared with CTR. (**G**) Dot plot showing enriched GO biological processes of DEGs between the striata of CTR and IS mice. (**H**) Dot plot showing enriched GO biological processes of DEGs between the striata of CTR and HS mice.

To investigate alterations in cellular diversity and molecular signatures following stroke, we analyzed a publicly available scRNA-seq data set from the striatum of control, HS, and IS mice (GSE167593). A total of 29,388 cells were classified into 10 distinct populations: astrocytes (*Gfap, Aldh1l1*), endothelial cells (*Flt1, Tek*), microglia (*Cx3cr1, Itgam, Aif1*), oligodendrocytes (*Mbp, Olig2*), T cells (*Cd4, Cd5, Cd3d, Trac*), neurons (*Rph3a, Chrna4, Pcdh20*), neuroblasts (*Dcx, Celf4, Crmp1*), neutrophils (*Slpi, Cebpe, Ltf*), ependymal cells (*1500015O10Rik, Ak7*), and macrophages (*Cd36, Mrc1*) (Fig 1A-1C). Comparative analysis revealed increased proportions of microglia, T cells, ependymal cells, and neutrophils in both HS and IS mice compared to controls (Fig, 1D-1E). While the expansion of microglia, T cells, and neutrophils reflects robust activation of the neuroimmune system, the elevated ependymal cell population may represent a compensatory response to blood-brain barrier (BBB) disruption, potentially contributing to BBB repair and inflammation containment [20, 21]. Accordingly, representative upregulated DEGs were associated with activation of disease-associated microglia (*Cst7*, *Apoe*) and BBB disruption (*Ccl5*, *Spp1*) (Fig. 1F) [22-24].

GO enrichment analysis of upregulated transcripts revealed that mitochondrial protein localization and mitochondria-related apoptosis dominated the enriched pathways following IS (Fig. 1G), while stress-activated MAPK cascade and the canonical NF-κB signaling pathway ranked highest following HS (Fig. 1H). Notably, both IS- and HS-upregulated genes were associated with mitochondrial autophagy and disassembly, suggesting the activation of compensatory mechanisms likely in response to persistent mitochondrial damage. This observation is consistent with previous reports of enhanced mitophagy and mitochondrial biogenesis post-stroke [25, 26].

**Figure 2.**
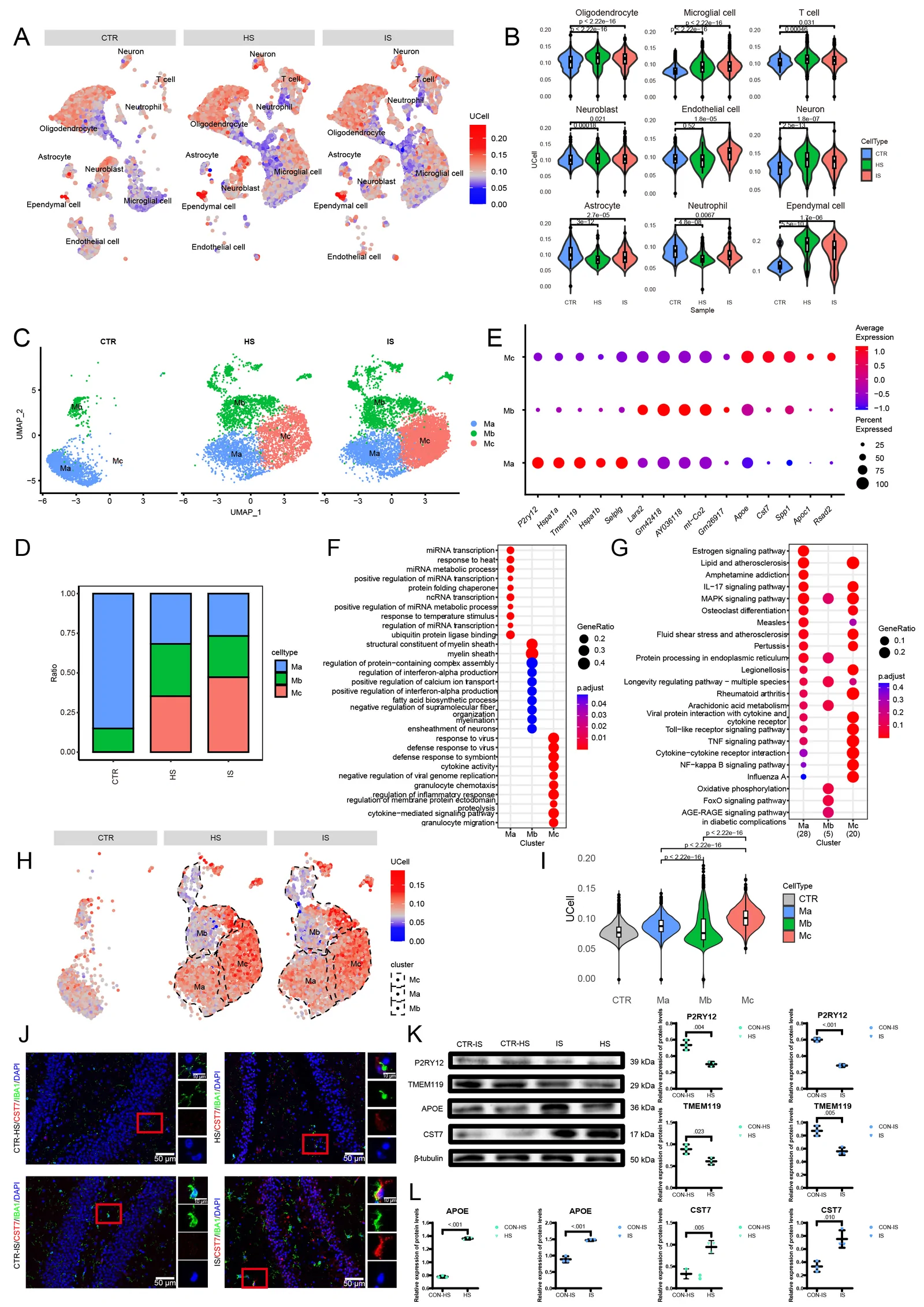
**Mitochondria-associated gene scoring identifies a strok-specific pro-inflammatory microglial population (Mc)**. (**A**) UMAP plot showing mitochondrial gene scores across cell types in the striata of CTR, HS and IS mice. (**B**) Violin plots of mitochondrial gene scores in different cell types from the striata of CTR, HS and IS mice. (**C**) UMAP plot demonstrating microglial subpopulations (Ma, Mb and Mc) identified in the three mouse groups. (**D**) Bar graph quantifying the proportions of microglial subpopulations shown in (C). (**E**) Dot plot showing marker gene expression patterns distinguishing Ma, Mb and Mc. (**F**) Dot plot showing enriched GO terms of biological processes associated with DEGs in Ma, Mb and Mc, respectively. (**G**) Dot plot showing enriched KEGG pathways associated with DEGs in Ma, Mb and Mc. (H and I) UMAP and violin plots displaying mitochondrial gene scores in Ma, Mb and Mc across CTR, HS and IS mice. (**J**) Representative immunofluorescence images of CST7 (red) and IBA1 (green) in ipsilateral (IS/HS) and contralateral (CTR-IS/CTR-HS) hippocampi of stroke mice. Scale bar, 50 µm; 10 μm for magnified insets. (K and L) Representative western blots and quantification of P2RY12, TMEM119, APOE, and CST7 in hippocampi described in (J). β-tubulin was used as a loading control. N=3 mice per group, indicating 3 biological replicates. Data normality was assessed using the Shapiro-Wilk test. Paired two-tailed Student's t-tests were used to determine the significance of difference between the ipsilateral and contralateral hippocampi. P values are shown in the figures.

### Stroke induces mitochondria-linked remodeling of microglial subtypes and a unique pro-inflammatory phenotype

Given the profound implication of mitochondrial pathology in the post-stroke brain, we next employed the UCell single-cell gene scoring tool to further understand primary mitochondria-associated genes affected by stroke. As expected, significant increases in mitochondrial gene scores across various cell types were observed in IS and HS brains compared to the control, indicating extensive involvement of mitochondria-related activities following stroke (Fig. 2A and 2B). Notably, microglia exhibited the most pronounced elevation in mitochondrial scores post-stroke, with a newly generated microglial population emerging (Fig. 2A). This suggests that stroke-induced mitochondrial pathology may influence microglial proliferation.

Subsequent clustering of microglial populations identified three transcriptionally distinct subclusters (Fig. 2C). The Ma subpopulation predominated in healthy mice but was markedly reduced in post-stroke (HS, IS) mice (Fig. 2D). This cluster highly expressed quiescence-associated markers, such as *P2ry12* and *Tmem119*, likely representing microglia at a resting state (Fig. 2E) [27, 28]. The Mb subpopulation exhibited elevated expressions of *Lars2* and *Gm42418* (Fig. 2E) and activation of the FOXO signaling pathway in response to stroke (Fig. 2F and 2G). Since FOXO activation promotes a shift toward an anti-inflammatory phenotype [29-32], Mb may represent anti-inflammatory microglia. Notably, the newly identified Mc subpopulation exhibited high expression of *Apoe* and *Cst7*, along with a genetic signature strongly linked to canonical pro-inflammatory signaling pathways, including IL-17, TNF, NF-κB, and Toll-like receptor cascades (Fig. 2E-2G) [33-36]. Therefore, Mc may be classified as pro-inflammatory microglia. In addition, among all microglial subsets, Mc displayed the highest alteration in mitochondrial scores following stoke (Fig. 2H and 2I), further supporting the association between mitochondrial dysfunction and pro-inflammatory microglial responses post-stroke.

To confirm the presence of the Mc subpopulation in the post-stroke brain, we performed immunofluorescence co-staining of IBA1, a classical marker of activated microglia, and CST7, a specific marker for Mc. Compared to the control mice, CST7 expression was more frequently found in IBA1+ microglia within the post-stroke brain tissue (Fig. 2J). Meanwhile, both HS and IS brains showed significant upregulation of CST7 and APOE and marked reductions in P2RY12 and TMEM119 (Fig. 2K and 2L), in line with our scRNA-seq results. These findings support the presence of stroke-specific Mc with a distinct *Cst7*^high^*Apoe*^high^*P2ry12*^low^*Tmem119*^low^ signature.

### LARS2 counteracts post-stroke pro-inflammatory microglial polarization by maintaining mitochondrial homeostasis

**Figure 3.**
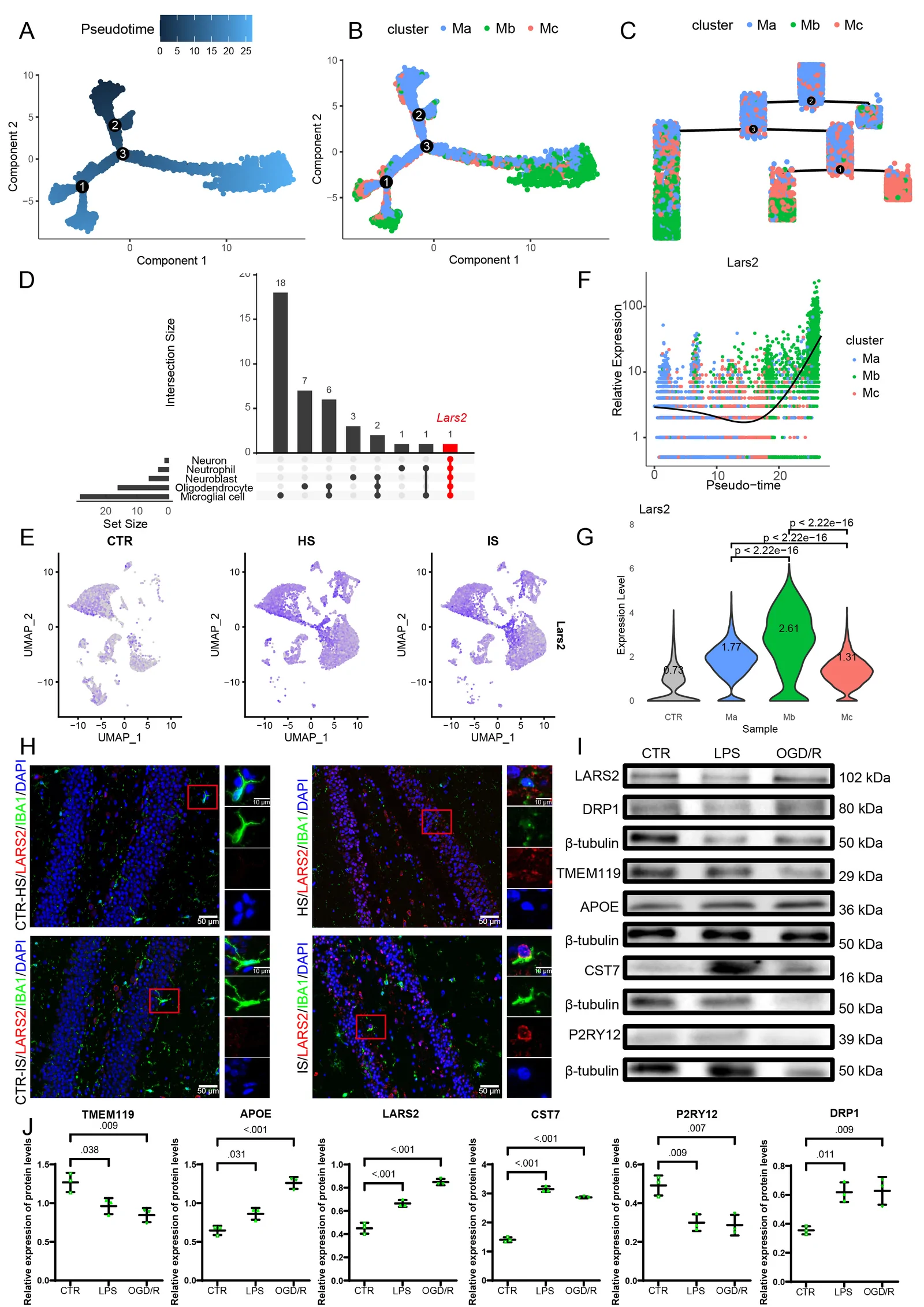
**Pseudotime analysis of developmental trajectories of microglial subtypes and validation of stroke-specific Mc**. (**A** and **B**) Pseudotime trajectory plots of microglia in stroke mouse brains without or with annotation of subpopulations (Ma, Mb and Mc). (**C**) Dendrogram illustrating the developmental relationships among stroke-affected migroglial subtypes (Ma, Mb and Mc). (**D**) UpSet plots depicting unique and overlapping mitochondria-related DEGs across cell types in IS and HS mouse striata. The dots indicate DEGs shared by different cell types. The bar graphs show the count of DEGs. (**E**) UMAP plots showing *Lars2* expression across the striata of CTR, HS, and IS mice. (**F**) Pseudotime trajectory plot of stroke-affected microglia integrated with *Lars2* expression profile. (**G**) Violin plot depicting *Lars2* expression in post-troke Ma, Mb and Mc compared to CTR microglia. (**H**) Representative immunofluorescence images of LARS2 (red) and IBA1 (green) in ipsilateral (IS/HS) and contralateral (CTR-IS/CTR-HS) hippocampi of stroke mice. Scale bar, 50 µm; 10 μm for magnified insets. (**I** and **J**) Representative western blots and quantification of LARS2, DRP1, TMEM119, APOE, CST7 and P2RY12 in BV2 cells treated with LPS or OGD/R and in untreated controls. BV2 cells were either stimulated with LPS for 6 h or subjected to oxygen-glucose deprivation for 2.5 h followed by reperfusion for 3 h (OGD/R). β-tubulin was used as a loading control. N=3 batches of cells per group, indicating 3 biological replicates. Data normality was assessed using the Shapiro-Wilk test. One-way ANOVA with Tukey's *post hoc* test was used to determine the significance of difference between groups. P values are shown in the figures.

To characterize the phenotypic plasticity of microglial subpopulations post-stroke, we performed pseudotime trajectory analysis on the sc-RNAseq data. Our analysis positioned the Ma subpopulation at the root of the trajectory, progressively diverging towards either the Mb or Mc phenotype (Fig. 3A-3C). Building on our previous findings implicating mitochondrial pathology in post-stroke microglial proliferation, we screened 39 mitochondria-associated DEGs between healthy and post-stroke microglia (Supplementary Fig. 4-6). Among these genes, *Lars2* exhibited the most pronounced regulation, significantly upregulated in stroke-affected microglia and broadly modulated across multiple cell types (Fig. 3D and 3E). Next, we overlapped the gene set positioned at the critical differentiation node (Node 3 in the trajectory) with the mitochondrial gene set to identify genes governing Ma fate decisions, revealing *Lars2* as a key nodal regulator. The pseudotemporal expression profile of *Lars2* exhibited a bimodal pattern: declining sharply during the Ma-to-Mc transition while markedly increasing along the Ma-to-Mb axis (Fig. 3F).

Accordingly, the Mb subpopulation showed the highest, the Mc subpopulation the lowest, and the Ma subpopulation intermediate *Lars2* expression levels (Fig. 3G). Moreover, LARS2 expression was found in activated migroglia (IBA1+) of stroke-affected brains but not in healthy control brains (Fig. 3H). These results suggest that *Lars2* may act as a central molecular rheostat orchestrating microglial differentiation and polarization.

To further investigate *Lars2*'s role in microglial activation, we treated BV2 cells with oxygen-glucose deprivation/reperfusion (OGD/R) or lipopolysaccharide (LPS) to mimick the microenvironment of ischemic stroke or neuroinflammation, respectively. As expected, both stimuli significantly reduced the expression of *Tmem119* and *P2ry12* and elevated *Cst7* and *Apoe* levels, marking microglial polarization from a resting to a pro-inflammatory phenotype (Fig. 3I and 3J). Notably, this transition was accompanied by the upregulation of LARS2 and the mitochondrial fission driver DRP1 (Fig. 3I and 3J). Since mitochondrial fragmentation is a hallmark of pro-inflammatory polarization and LARS2 plays a critical role in maintaining mitochondrial structural integrity [27-31], it is reasonable to infer that the upregulation of LARS2 represents an emergency measure to conteract microglial activation and pro-inflammatory polarization post-stroke, in line with our observation of a global increase in LARS2 levels in stroke-affected microglia.

Interestingly, it has been reported that LARS2 enhances the activity of E2F1, a transcription factor that directly binds to the *Mfn2* promoter and thereby influences mitochondrial dynamics by driving *Mfn2* expression [8]. We next evaluated this regulatory mechanism in pro-inflammatory microglia *in vitro*. Our results demonstrated that *Lars2* knockdown in OGD/R- or LPS-treated BV2 cells led to significant decreases in both E2F1 and MFN2 expression (Supplementary Fig. 7A-7C), with MFN2 downregulation resembling that observed following *E2f1* knockdown (Supplementary Fig. 7D and 7E). Conversely, *Lars2* overexpression restored the expression of both genes (Supplementary Fig. 7A-7C). Moreover, ChIP-qPCR assays confirmed that E2F1 binds to the *Mfn2* promoter in BV2 cells (Supplementary Fig. 7F). Altogether, these results suggest that dysregulated LARS2 disturbs mitochondrial dynamics and may contribute to the pro-inflammatory transition of microglia following stroke.

### Inhibition of *Lars2* expression induces post-stroke mitochondrial defects and the emergence of Mc *in vitro*

**Figure 4.**
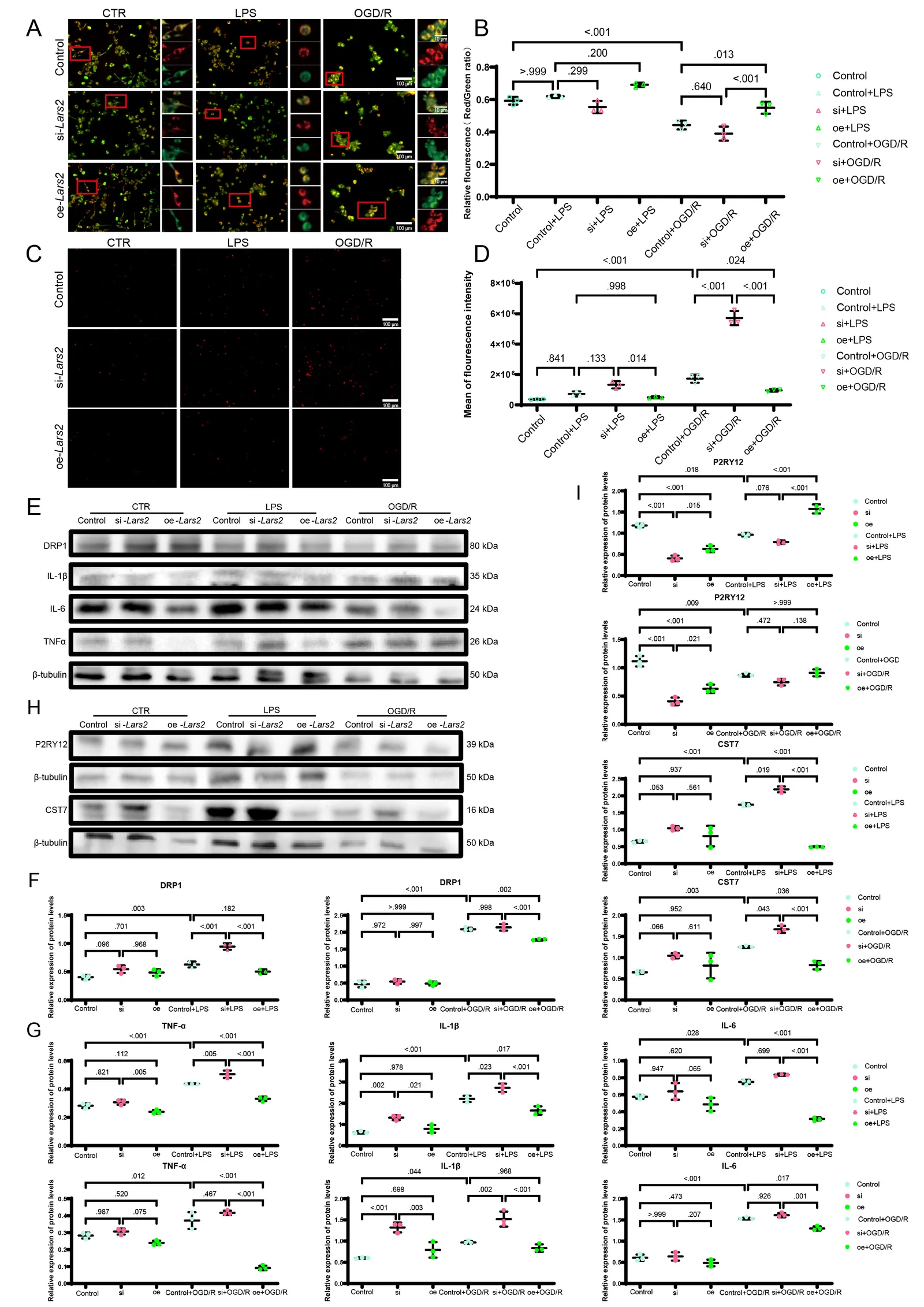
**LARS2 protects mitochondrial integrity and suppresses the pro-inflammatory phenotype of stroke-stressed BV2 cells**. (**A** and **B**) Representative immunofluorescence images and quantification of mitochondrial membrane potential in uninduced (Control) and LPS- or OGD/R-induced BV2 cells following *Lars2* knockdown (si+LPS, si+OGD/R) or overexpression (oe+LPS, oe+OGD/R) or respective controls (Control+LPS, Control+OGD/R). The JC-1 dye was used to evaluate mitochondrial membrane potential. Scale bar, 100 μm; 10 μm for magnified insets. (**C** and **D**) Representative immunofluorescence images and quantification of mtROS in the seven groups of BV2 cells described in (B). mtROS levels are represented by the average fluorescence intensity of MitoSOX™ labeling. Scale bar, 100 μm. (**E-I**) Representative western blots and quantification of DRP1, IL-1β, IL-6, TNF-α, CST7 and P2RY12 in uninduced (Control) and LPS- or OGD/R-induced BV2 cells following *Lars2* knockdown (si+LPS, si+OGD/R) or overexpression (oe+LPS, oe+OGD/R). N=3 batches of cells per group, indicating 3 biological replicates. Data normality was assessed using the Shapiro-Wilk test. One-way ANOVA with Tukey's *post hoc* test was used to determine the significance of difference between groups. P values are shown in the figures.

To further determine whether post-stroke microglial polarization was driven by LARS2-mediated mitochondrial dysfunction, we evaluated the impact of *Lars2* expression alteration in OGD/R- or LPS-induced BV2 cells. JC-1 and mtROS assays revealed that *Lars2* knockdown significantly exacerbated mitochondrial impairment, evidenced by decreased mitochondrial membrane potential and increased mtROS production, whereas *Lars2* overexpression ameliorated these deficits (Fig. 4A-4D). On one hand, *Lars2* knockdown upregulated *Drp1*, while its overexpression suppressed *Drp1* expression (Fig. 4E and 4F). On the other hand, *Lars2* knockdown enhanced the expression of pro-inflammatory cytokines TNF-α, IL-1β, and IL-6, which were attenuated upon *Lars2* overexpression (Fig. 4E-4G). Furthermore, *Lars2* knockdown increased the expression of the Mc marker CST7, while decreasing the resting microglia marker P2RY12, and these effects were both reversed by *Lars2* overexpression (Fig. 4H and 4I). Collectively, these findings establish *Lars2* as a critical regulator of mitochondrial function and microglial phenotype post-stroke.

### The SDC4-PTN receptor-ligand axis mediates communication between Mc and neurons

**Figure 5.**
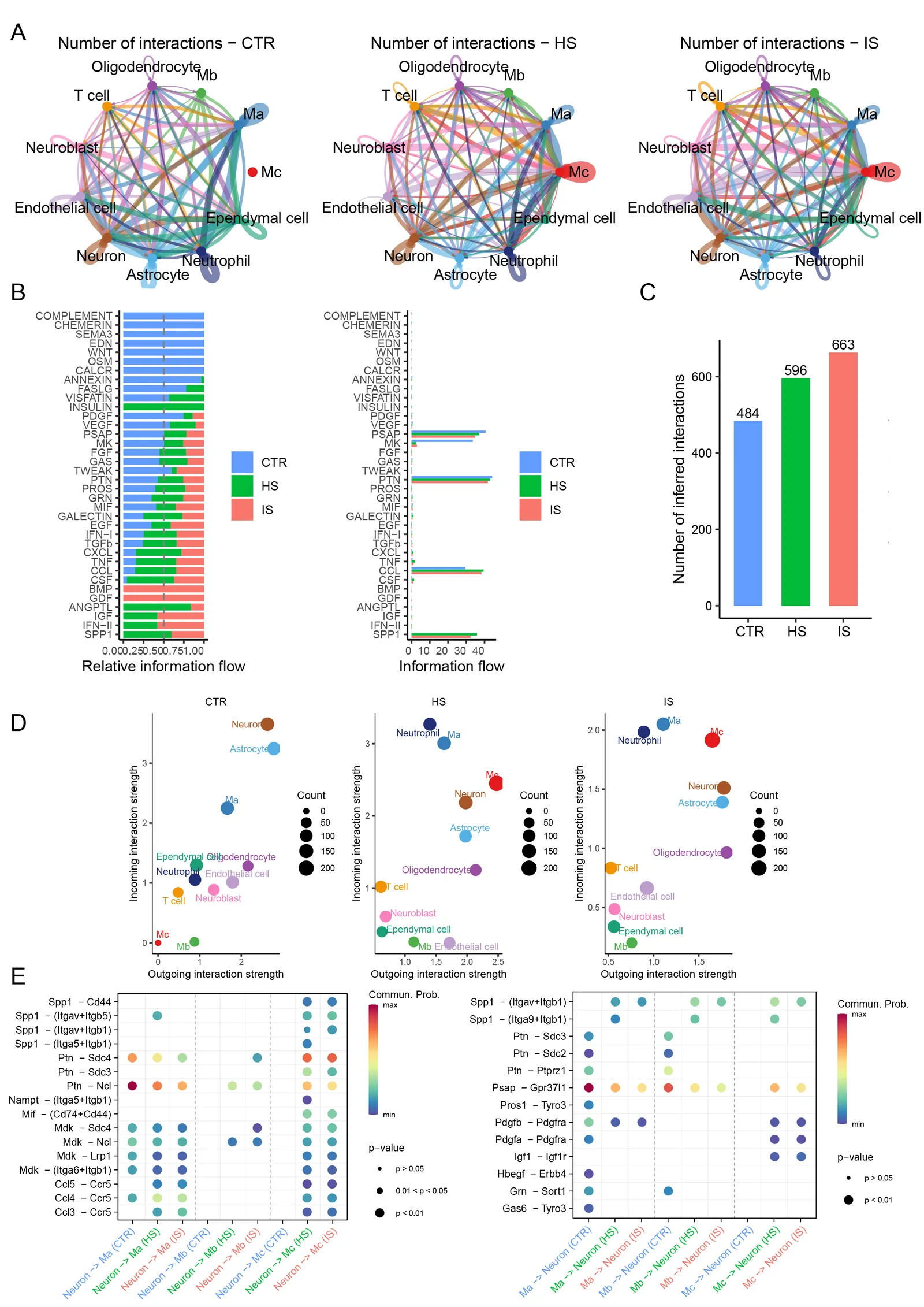
**Intercellular interactions in the striatum are significantly altered in HS and IS mice compared to controls**. (**A**) Interaction networks among different cell types in the striata of CTR, HS and IS mice. The nodes represent cell types with the node size showing the interaction frequency. The edges represent intercellular interactions with the edge thickness and transparency reflecting the interaction strength. (**B**) Bar graphs showing the proportions of enriched signaling pathways in the striata of the three mouse groups. (**C**) Bar graph displaying the number of inferred intercellular interactions in the striata of the three mouse groups. (**D**) Dot plots illustrating the incoming and outgoing interaction strengths of various cell types in the striata of the three mouse groups. (**E**) Dot plots demonstrating ligand-receptor pairs between microglial subpopulations (Ma, Mb and Mc) and neurons in the striata of the three mouse groups.

Considering that cell-cell communication mediated by ligand-receptor interactions is fundamental to the regulation of both physiological and pathological processes, we next focused on how these interactions are reshaped after stroke. Significantly, a marked increase in both the number and strength of inferred interactions was observed in stroke-affected brains, involving activation of multiple cascades including the CCL and SPP1 signaling pathways (Fig. 5A-5C). Among the three microglial subpopulations, Mc exhibited the most extensive interactions with other cell types (Fig. 5D).

Particularly, the pleiotrophin-syndecan-4 (PTN-SDC4) ligand-receptor pair, previously known to facilitate macrophage inflammation and invasiveness but underexplored in the context of stroke [32-36], was identified as a key mediator of communication between neurons and Mc (Fig. 5E).

**Figure 6.**
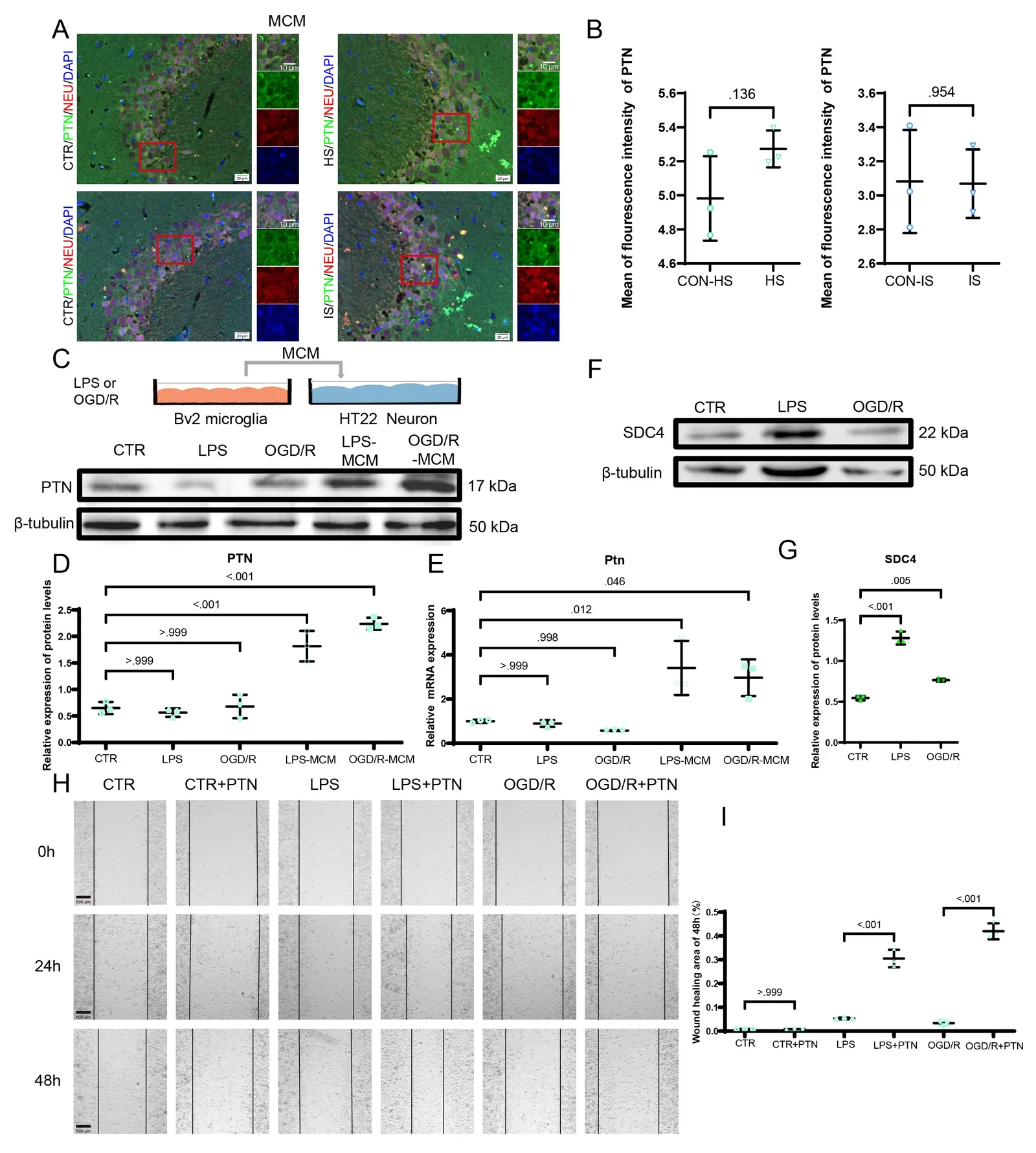
**The PTN-SDC4 receptor-ligand axis mediates communication between pro-inflammatory microglia and neurons following stroke**. (**A** and **B**) Representative immunofluorescence images and quantification of PTN (green) and NEUN (red) in ipsilateral (IS/HS) and contralateral (CTR-IS/CTR-HS) hippocampi of stroke mice. Scale bar, 50 µm; 10 μm for magnified insets. (**C** and **D**) Representative western blots and quantification of PTN in HT22 cells treated with LPS, OGD/R or conditioned media from LPS- or OGD/R-induced BV2 cells (LPS-MCM or OGD/R-MCM) and untreated controls (CTR). (**E**) mRNA levels of *Ptn* in the five groups of HT22 cells described in (D). (**F** and **G**) Representative western blots and quantification of SDC4 in uninduced (CTR) and LPS- or OGD/R-induced BV2 cells. (**H** and **I**) Scratch assay and migration analysis of uninduced and LPS- or OGD/R-induced BV2 cells with (CTR+PTN, LPS+PTN, OGD/R+PTN) or without (CTR, LPS, OGD/R) exogenous PTN supplementation. BV2 cells were treated with PTN (30 ng/mL) alone or in combination with LPS or OGD/R for 48 h. Scratch assays were performed at 0 h, 24 h, and 48 h. The wound healing areas of BV2 cells in different cell groups were quantified to assess migration distances. Scale bar, 500 µm. In (D) and (G), β-tubulin served as loading control. For (B),N=3 mice per group; for (D-G) and (I), N=3 batches of cells per group, indicating 3 biological replicates. Data normality was assessed using the Shapiro-Wilk test. The statistical significance was determined using paired two-tailed Student's t-tests for (B) and one-way ANOVA with Tukey's *post hoc* test for (D-G) and (I). P values are shown in the figures.

Interestingly, although PTN expression remained unchanged in NEUN+ neurons from stroke-affected brains and in HT22 neuronal cells treated with OGD/R or LPS (Fig. 6A-6E), exposure to conditioned medium of BV2 microglial cells treated with OGD/R or LPS significantly upregulated PTN expression in HT22 cells (Fig. 6C-6E). In parallel, we observed upregulated SDC4 expression in those OGD/R- or LPS-treated BV2 cells (Fig. 6F and 6G). In addition, exogenous PTN supplementation enhanced the migration of OGD/R- or LPS-treated BV2 cells but not that of untreated controls, as shown in the scratch wound assay (Fig. 6H and 6I). These findings suggest that the PTN-SDC4 receptor-ligand axis underlines communication between pro-inflammatory microglia and neurons following stroke. Whether this interaction leads to excessive microglial phagocytosis of neurons and contributes to neuroinflammation warrants further investigation.

### LARS2 expression declines with aging

To investigate whether LARS2 expression is altered during aging, a process involving neuroinflammatory changes often regulated by the hypothalamus, we analyzed a publicly available scRNA-seq data set (GSE188646) derived from young and aged mouse hypothalamus. Significant reductions in *Lars2* expression were observed across multiple cell types in aged mouse hypothalamus compared to their younger counterparts (Supplementary Fig. 8A and 8B). Strikingly, *Lars2* expression was undetectable in microglia of aged mice (Supplementary Fig. 8B). We further examined *LARS2* expression patterns in human microglia by integrating several published scRNA-seq datasets (GSE157827, GSE160936, GSE148822, GSE140231, GSE163737, GSE186538, and GSE212606). Our analyses revealed that *LARS2* expression in microglia peaked at around age 59, followed by a gradual decline thereafter (Supplementary Fig. 9). These observations indicate that LARS2 downregulation is a conserved feature of aging microglia across species and brain subregions, potentially relevant to age-associated brain changes.

## DISCUSSION

In this study, we identify and characterize a stroke-specific pro-inflammatory microglial subpopulation (Mc) associated with mitochondrial dysfunction. We demonstrate that post-stroke mitochondrial damage triggers a compensatory upregulation of *Lars2*, a critical gene for maintaining mitochondrial integrity. However, Mc microglia exhibit insufficient *Lars2* expression, impairing their capacity to restore mitochondrial function. This deficiency perpetuates mitochondrial oxidative stress and promotes the pathological polarization of microglia towards a pro-inflammatory phenotype. Importantly, *Lars2* overexpression restores mitochondrial integrity and suppresses microglial polarization, underscoring its therapeutic potential in stroke recovery.

The Mc subpopulation, characterized by elevated *Cst7* and *Apoe* and reduced *P2ry12*, partly overlaps with the transcriptional profile of a disease-associated microglia (DAM) subtype identified in Alzheimer’s disease (AD) [14]. While DAM are linked to TREM2 signaling-dependent amyloid-β phagocytosis and considered beneficial in slowing AD progression [37-39], Mc appear to arise primarily in response to impaired mitochondrial quality control and may instead contribute to neuroinflammation. Notably, mitochondrial dysfunction marked by disruption of electron transport chain complexes I, III, and IV, or mitochondrial DNA depletion has been shown to augment *Apoe* expression [40, 41]. Additionally,*Cst7* upregulation may counteract mtROS-induced lysosomal permeability, and subsequent cell death [42-53]. These findings suggest that *Cst7* and *Apoe* may serve not only as biomarkers for specific microglial populations but also as indicators of cellular responses to mitochondrial stress. Collectively, our results, along with those of others, underscore the functional diversity of microglial subpopulations and highlight the context-dependent roles they play across different neurological diseases.

**Figure 7.**
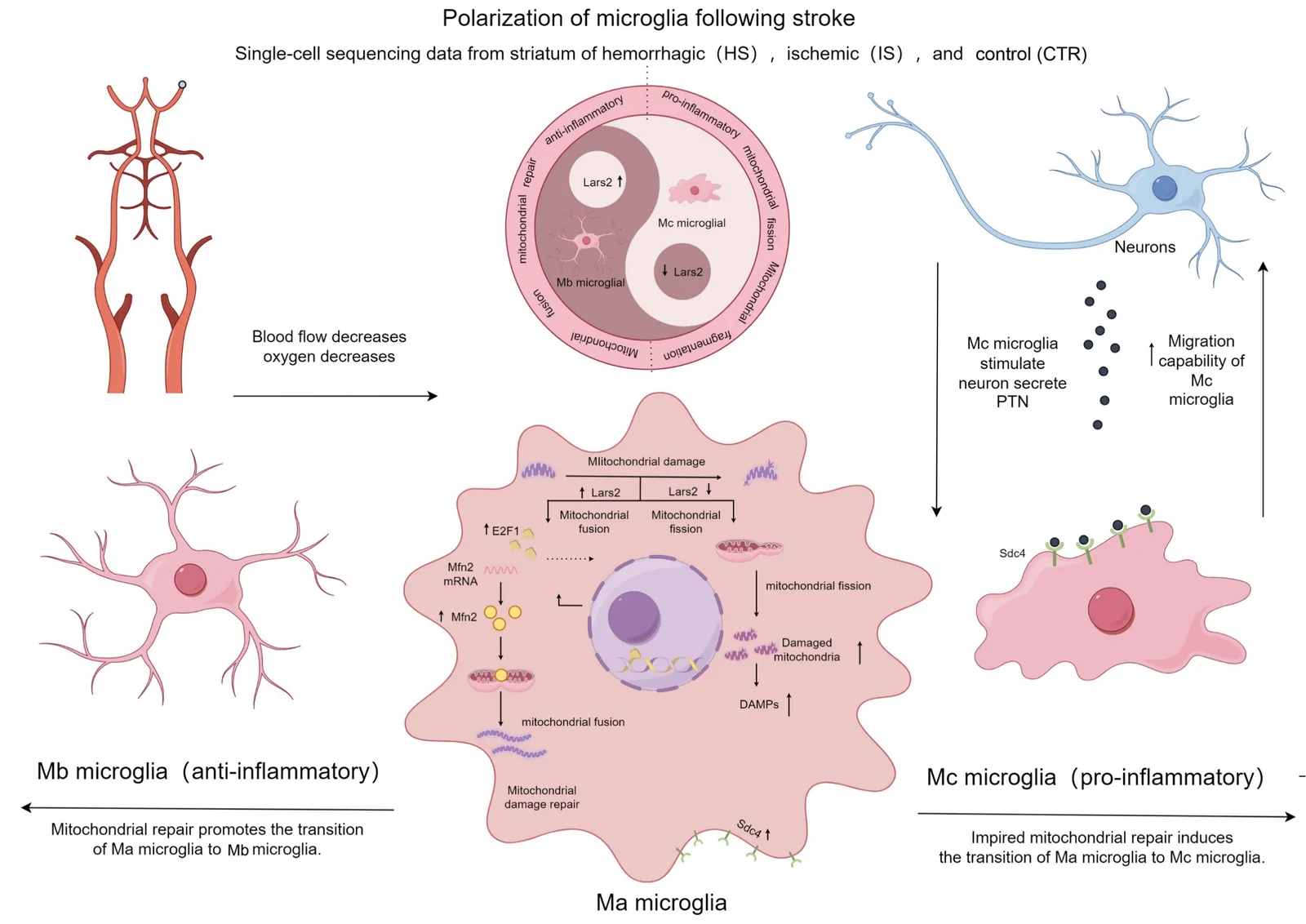
**Schematic diagram illustrating the mechanism of pro-inflammatory microglial polarization following stroke**. During stroke, microglia undergo a phenotypic transition towards either the Mb anti-inflammatory or Mc pro-inflammatory state. In the acute response, LARS2 promotes the upregulation of E2F1, which in turn increases MFN2 expression. This cascade facilitates the fusion of damaged mitochondria, thereby reshaping the mitochondrial network and driving microglial polarization towards Mb, representing a compensatory mechanism. In microglia lacking sufficient self-repair capacity, LARS2 expression is diminished, leading to the suppression of MFN2-mediated mitochondrial fusion. Instead, DRP1-drived mitochondrial fission predominates, resulting in the accumulation of damaged mitochondria, which release DAMPs and thereby promote migroglial transition to the pro-inflammatory Mc phenotype. Activated Mc subsequently stimulates neurons to secrete PTN, which binds back to SDC4 receptors on Mc. This ligand-receptor interaction enhances Mc migration toward neurons and exacerbates post-stroke neuroinflammation.

In the context of stroke, we propose a *Lars2-E2f1-Mfn2* axis that regulates mitochondrial remodeling (Fig. 7). Damaged mitochondria have been shown to recruit the NF-κB essential modulator (NEMO), thereby activating the IκB kinase (IKK) complex [54]. This activation in turn triggers nuclear translocation of NF-κB, a central mediator of pro-inflammatory responses [54]. Interestingly, while a previous study reported that MFN2 is required for NF-κB activation and subsequent pro-inflammatory macrophage polarization [55], our findings reveal a contrasting role of MFN2 in microglia, where MFN2, regulated by LARS2, may instead restrain pro-inflammatory activation.

Pro-inflammatory microglial polarization is often accompanied by increased *Sdc4* expression [56-59]. Notably, the *Sdc4* promoter contains a binding site for the NF-κB subunit p65 (RelA) [60], supporting its regulation by NF-κB signaling. Given that LARS2 modulates mitochondrial homeostasis and may suppress NF-κB activity through MFN2, it is plausible that LARS2 downregulates *Sdc4* expression by interfering with NF-κB activation. Importantly, as we show that the interaction between HT22 neuronal cells and BV2 microglial cells are strengthened via the PTN-SDC4 ligand-receptor pair, the *Lars2-E2f1-Mfn2* axis could in turn influence neuron-microglia communication, especially under post-stroke inflammatory conditions. However, whether this enhanced interaction leads to abnormal neuronal phagocytosis and contributes to excessive neuronal loss remains unknown. Further studies are needed to elucidate this potential mechanism.

Beyond stroke, mitochondrial dysfunction is a defining feature of aging, characterized by impaired mitochondrial oxidative phosphorylation, elevated oxidative stress, and altered dynamics [61]. These changes contribute to inflammaging and increased vulnerability to neurological insults, including stroke. Restoration of mitochondrial ultrastructure has been shown to suppress inflammation driven by damage-associated molecular patterns (DAMPs) released from senescent mitochondria [62-65]. Our study identifies LARS2 as a key regulator of mitochondrial homeostasis in microglia. The observed aging-associated decrease in *Lars2/LARS2* expression, particularly in microglia, may impair mitochondrial quality and lower the threshold for inflammatory activation, thereby exacerbating neuroinflammation in the aged brain following stroke. These findings not only highlight *LARS2* as a potential molecular link between stroke severity and aging but also points to the possibility that targeting *LARS2*-mediated mitochondrial regulation may offer therapeutic strategies to mitigate age-related neuroinflammatory damage.

## Supplementary Materials

The Supplementary data can be found online at: www.aginganddisease.org/EN/10.14336/AD.2025.0387.



## Data and materials availability

The sc-RNAseq data analyzed in the present study are publicly available in the Gene Expression Omnibus (GEO) repository under accession numbers GSE167593, GSE188646, GSE157827, GSE160936, GSE148822, GSE140231, GSE163737, GSE186538, and GSE212606. Other data reported in this manuscript and supplementary files are available from the corresponding authors upon reasonable request.
