# Supplementary material for: Lars2 Deficiency-Induced Mitochondrial Dysfunction Drives the Emergence of a Pro-Inflammatory Stroke-Specific Microglial Subpopulation
Source: Aging Dis. 2025 Jun 16;17(4):2213–30. doi: 10.14336/AD.2025.0387 (PMC13256556; doi:10.14336/AD.2025.0387)
Supplement: Supplementary file 1 [file AD-17-4-2213-s.pdf]

SUPPLEMENTARY DATA

Lars2 Deficiency-Induced Mitochondrial Dysfunction Drives the Emergence of a Pro-Inflammatory Stroke-Specific Microglial Subpopulation

Qing Zou, Jianxin Zhou, Ying Li, Jiaming Shi, Jingying Huang, Cheng Zhuang, Hao Wu, Huanle Hong, Yanan Guo, Qian Li, Robert Chunhua Zhao, Jiao Wang

© 2025. Zou Q et al. Published online at http://www.aginganddisease.org/EN/10.14336/AD.2025.0387

SUPPLEMENTARY DATA

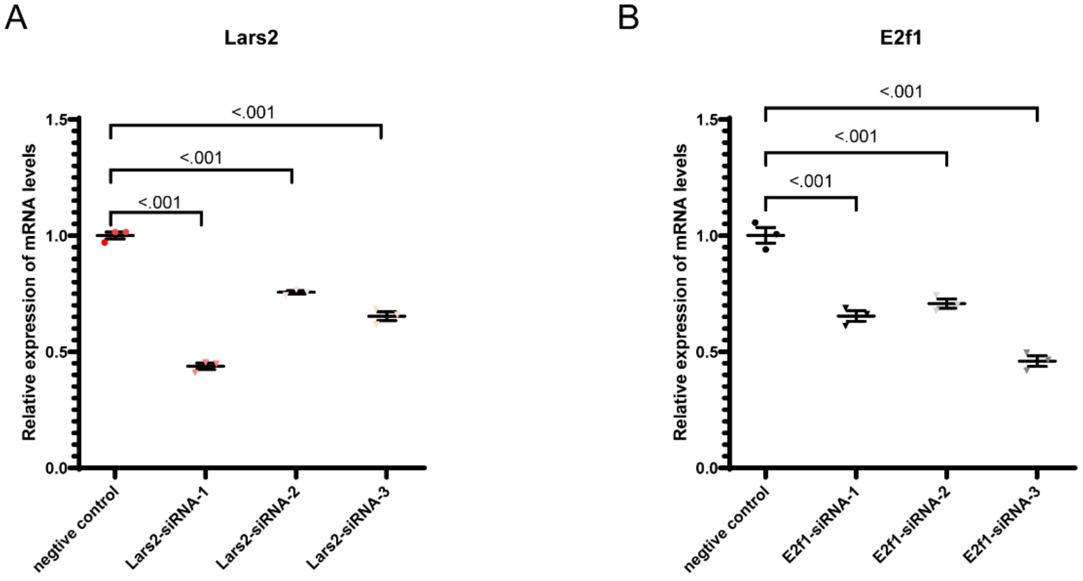

Supplementary Figure 1. Silencing efficiency of *Lars2* and *E2f1* siRNAs in BV2 cells. (**A**) mRNA levels of *Lars2* in BV2 cells transfected with control siRNA (negative control), *Lars2*-siRNA1, *Lars2*-siRNA2, or *Lars2*-siRNA3. (**B**) mRNA levels of *E2f1* in BV2 microglial cells transfected with control siRNA (negative control), *E2f1*-siRNA1, *E2f1*-siRNA2, or *E2f1*-siRNA3. N=3 batches of cells per group, indicating 3 biological replicates. Data normality was assessed using the Shapiro-Wilk test. One-way ANOVA with Tukey's *post hoc* test was used to determine the significance of difference between groups. P values are shown in the figures.

© 2025. Zou Q et al. Published online at http://www.aginganddisease.org/EN/10.14336/AD.2025.0387

SUPPLEMENTARY DATA

>NC_000070.7:c147989528-147991527 Mus musculus strain C57BL/6J chromosome 4, GRCm39
AAGTTTCCTGTGTAATCCAGGCTGGCCTCTCACCTGTGATCCTCCTGCCTCAACCCCTGAG-
TACTGGGATGACTGACATGAACCGTCATGTCCTGTCCAGGGTTGTTTCTAATAGTGACCATTTTC
TGGTATGTGCAATTCATATACACAGTGGAAAAGTCGATCAAAACAGGAATAACTGACATAAGAAA
AGCCCCCTGCAGTTGAACCCCAGTACTCGCTAGACCCATTTCTAGTGGCAGTCTGCAGTACTG
GAGGAACCTTGCCATTGAGGGGTTAAAGGAAGAGCCTCACAGTTTGGAGTTCTGGTTTAGGCA
AGCCCAAGTGGAATGTAAGATTAGAAGCCTGGGAATGGGCTCAGAAGTTCCCTCTTCTGTCCA
GGTTGGGATGGGAGCCTAGGCCAAAGCTTTCAGGGAGGCT**ACAGCAGAGGCAGAATGGACA**
GGAGAGAGTTTGGGAAAGCTCCTCCAAGCCTCTGGCTTGAGTAAGATTCTGCCAAAGGGAA
AGGAAACAGTCTTCAGTTTTGTACTCAAAGAATTCCCTGGGGAGGCTGGAGATGATGGAGGA
GGAGATACTTAGCGCACACTATACCCCAAGGCAAGTGCTGGGTGTGTCTCTCCAGTTCAGAG
TCAGATGTTCCAGACAAGAAGCTGCCCTATGGGCAGTGACTACAGGCCAGCTCCCATGACTCA
CCTCTCTGGAGACTCGTAACCGGCGTGCTGATTTACCTCTGAGGCTGTTTGTGGCATGACTGTA
CCCTGGCTATAGGGCAGTGCCAATCGGTAGAAAGCATAACATGGCAGCTAGGTGTGTCAGTGA
GATGACTACAGTCCTAGCCCTTGGGAGGCAGAGTAAAAAGATCATCAATTCAAGGCTGGTTTGG
GCTACATAGTGGGAAGCCTGCCCCCAAACAAACAAAAGCAAGGTCGCATGGGCCACAGACACA
GTTAAAATATTCAGCTAGCCATATTATCTGCTTTTAAATACTTGGCTTGTTGTTTTTATTTAATGTGT
ATGGGATGCTGCCTGCATGTATGGAAGTGTATCAGAAGTGTGCCTGGTTCCCCCTGGAAGACA
GAGAGGACACTGGATCCCCTGAATTGTAGTTACCGATGTGAGCTAACATGTAGGTGTTGGGTCC
TCTGTAAAAGCCATCTCTCTAGGCCCCAACCATATTCATTTATCGTGTGTGGTTGATGTACACGT
GCCCTGGCTCTTGTACGGAGGTTAGAGGACAACATTTTATGAGCAGGTTCTTTCCTTCCACCGT
GTGGGTTCCTGGAGATGGAACTCAAGCTGTCAGGCTTGGTAGCAGCCATTTATACCTACTGAGC
CTTTTTTGTGACCGCCACTATTAGCTCTACTTTTAGAAGTAAGAAGAAACGGGGTGGGGGGGGC
GATTTAATTAAGATAATGTATTTCATTTAGCCCAGTTTATCCAAAGCTATAACAACACATCAATATAA
AATTACTAATAAGGTAGTTTGCATTTCTTTTTATATTAAACATTGGGGCATTTCTCAGTTTGTAGCA
GCTGCATTTCAGGGTCTCAGTAGCTACAAGTGGCTGGTGGGTGTTACTACAGAGCCCAGGCTT
GGAGCCCTCCCAGCTTGGGGTCCAATGCAGTATCCCAGTTCTTGGAACAAAATGAGGGGCCAG
TGAATGGCATGTGAGGTTGGATAATCATGTCCTGAATGTCCTGGGACTGAGTGAAAATTAAAAAC
TGCATGTTAGATAGCCAGTGCAATGACTGTCACCTAGTAGCTGCCTGATGCGTTGTGGCTGCTG
TTTAGTTGCCTCAAAGGCAACTGAAGGACAATTACTTCACCCTAACGGTCTGTTTGTTCCCTTTG
AAGACACTCGGATTATGAAATGCGAGTCTGGATTTCAAAACTGACAGGTGTTATACAGGGTCAAA
TTCTGGAGCAGCAACAACAAAAAAGGCGCAACAACAAAAAAGGCGCA

Supplementary Figure 2. Amplified sequence of *Mfn2* promoter detected by CHIP-qPCR. The yellow-highlighted sequence indicates the specific *Mfn2* promoter fragment amplified by CHIP-qPCR.

© 2025. Zou Q et al. Published online at http://www.aginganddisease.org/EN/10.14336/AD.2025.0387

SUPPLEMENTARY DATA

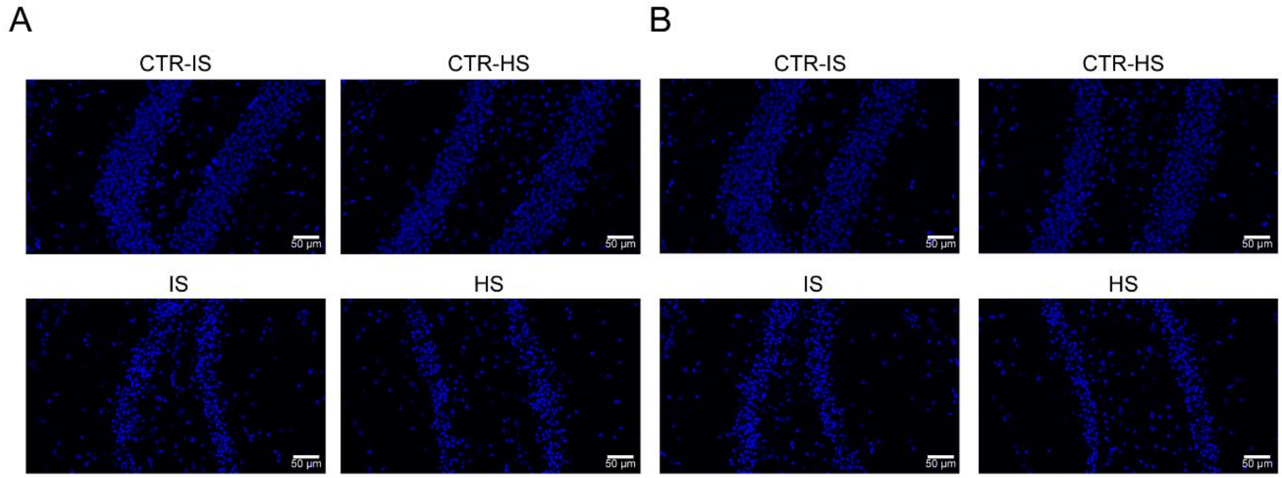

Supplementary Figure 3. Negative controls for immunofluorescence staining. (**A**) Representative immunofluorescence images of isotype controls using anti-rabbit IgG (red) and anti-mouse IgG (green) in the ipsilateral (IS/HS) and contralateral (CTR-IS/CTR-HS) hippocampi of stroke mice. (**B**) Representative immunofluorescence images of secondary antibody-only controls using Alexa Fluor 488-conjugated goat anti-rabbit IgG (green) and Alexa Fluor 594-conjugated goat anti-mouse IgG (red) in the hippocampi of stroke mice. Scale bar, 50 µm.

© 2025. Zou Q et al. Published online at http://www.aginganddisease.org/EN/10.14336/AD.2025.0387

SUPPLEMENTARY DATA

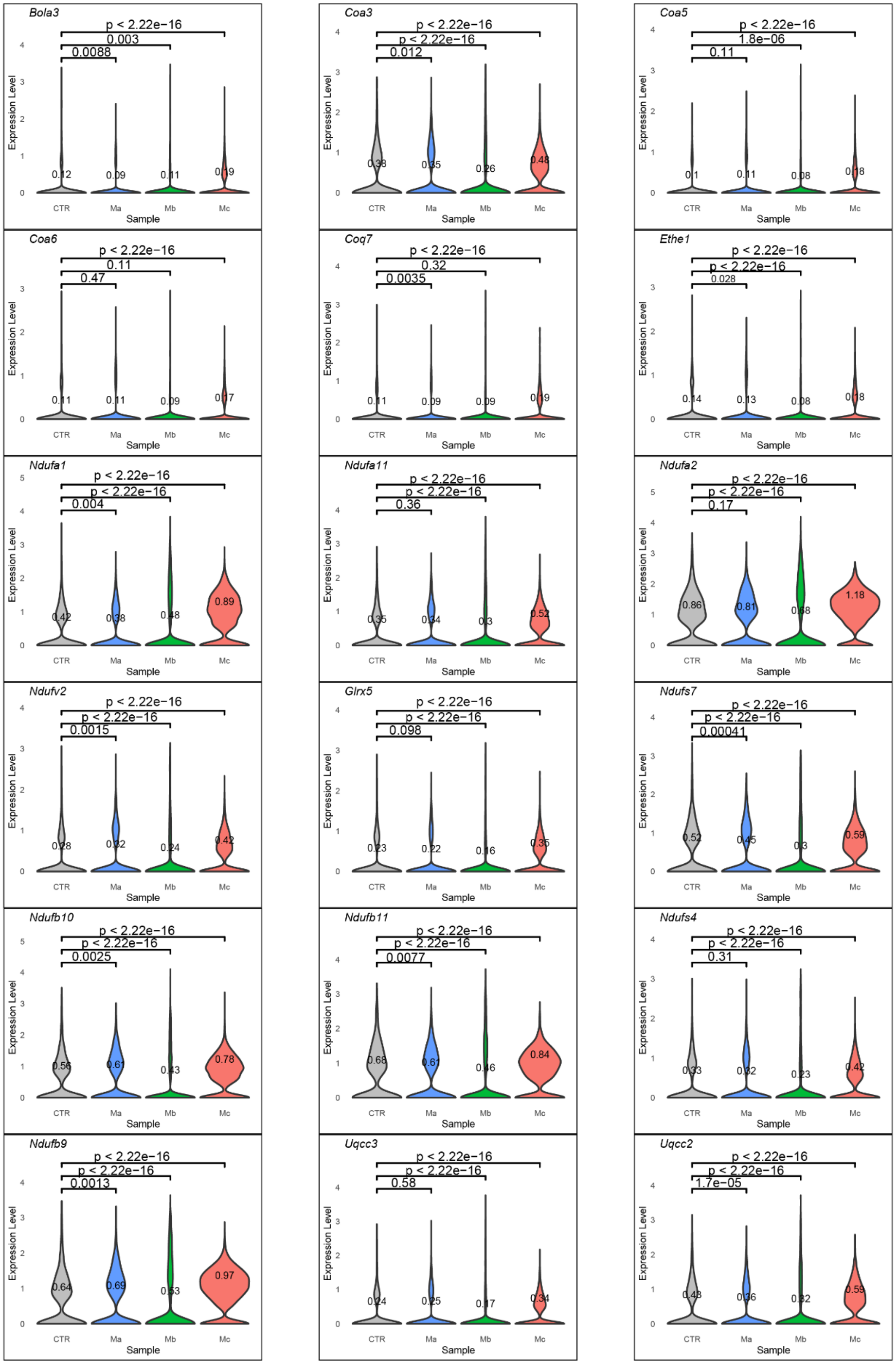

Supplementary Figure 4. Violin plots (Part I) illustrating the differential expression of representative mitochondria-related genes across the three post-stroke microglial subpopulations (Ma, Mb and Mc) compared to microglia from the control group.

© 2025. Zou Q et al. Published online at http://www.aginganddisease.org/EN/10.14336/AD.2025.0387

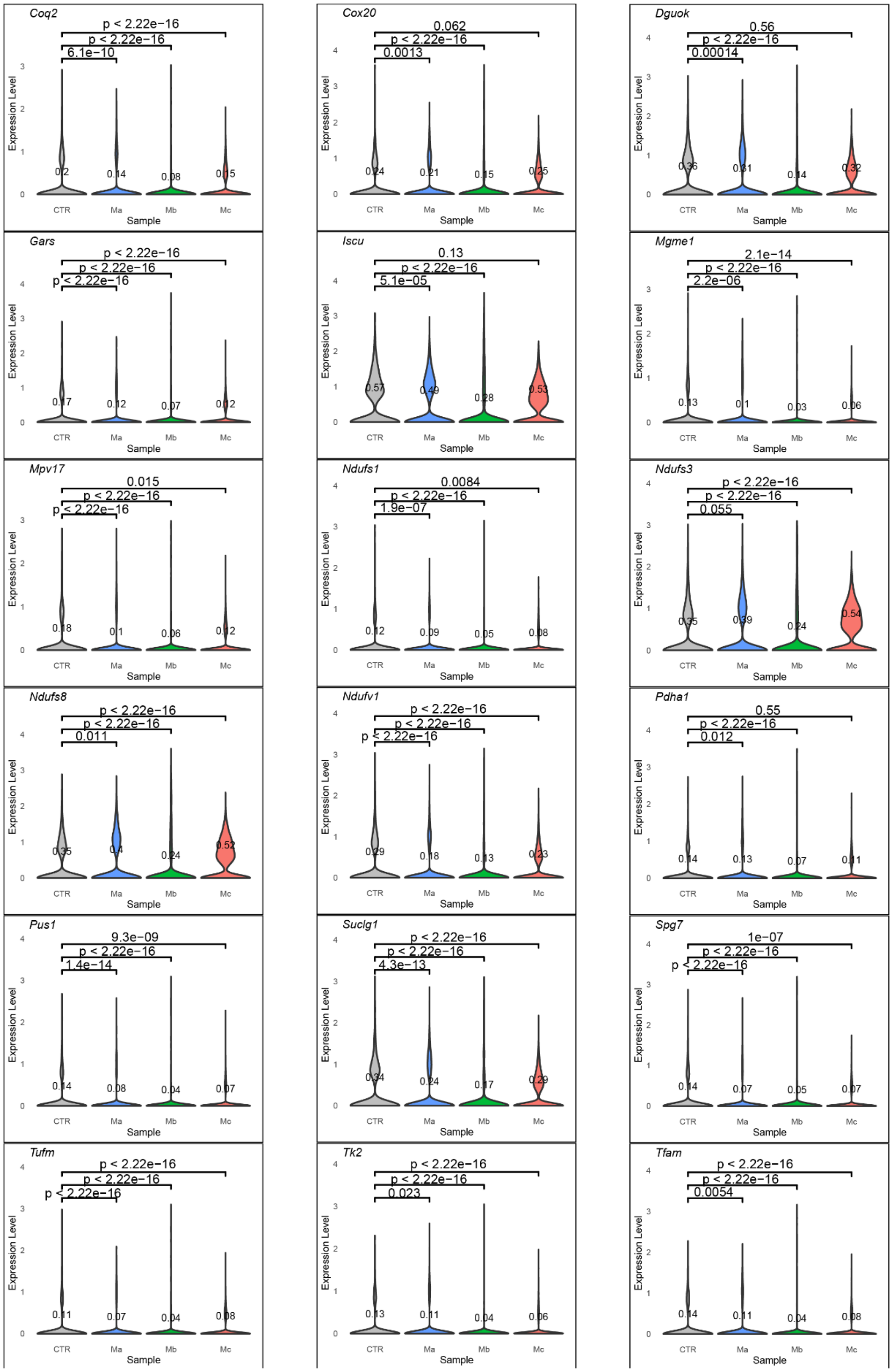

Supplementary Figure 5. Violin plots (Part II) illustrating the differential expression of mitochondrial-related genes across the three microglial subpopulations (Ma, Mb and Mc), compared to microglia from the control group.

SUPPLEMENTARY DATA

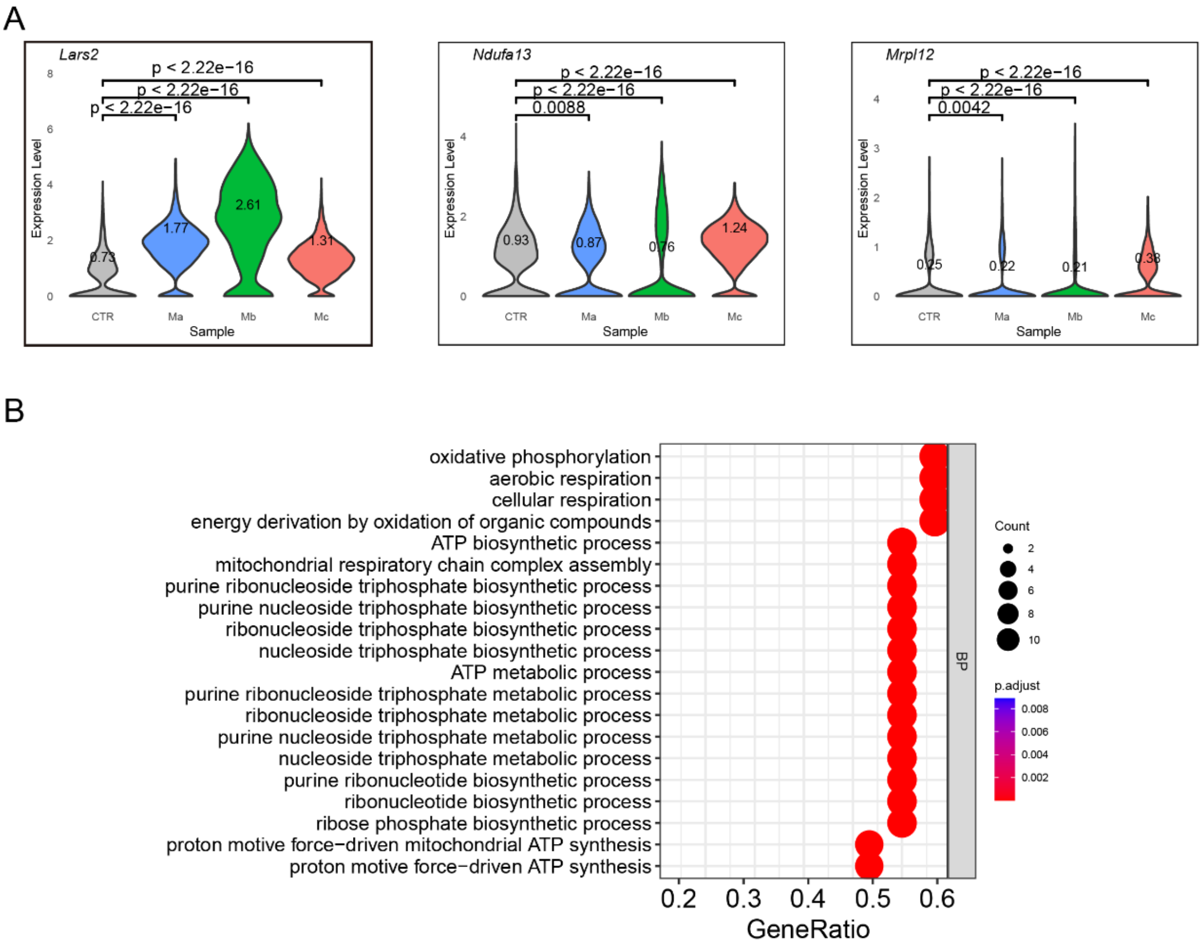

Supplementary Figure 6. Expression levels of representative mitochondria-related DEGs in stroke-affected microglia and functional enrichment analysis. (**A**)Violin plots depicting the expression levels of *Lars2*, *Ndufa13*, and *Mpl12* in post-stroke microglial subpopulations (Ma, Mb and Mc) and control microglia (CTR). (**B**) Dot plot showing enriched GO biological processes associated with mitochondria-related DEGs between control and post-stroke microglia.

© 2025. Zou Q et al. Published online at http://www.aginganddisease.org/EN/10.14336/AD.2025.0387

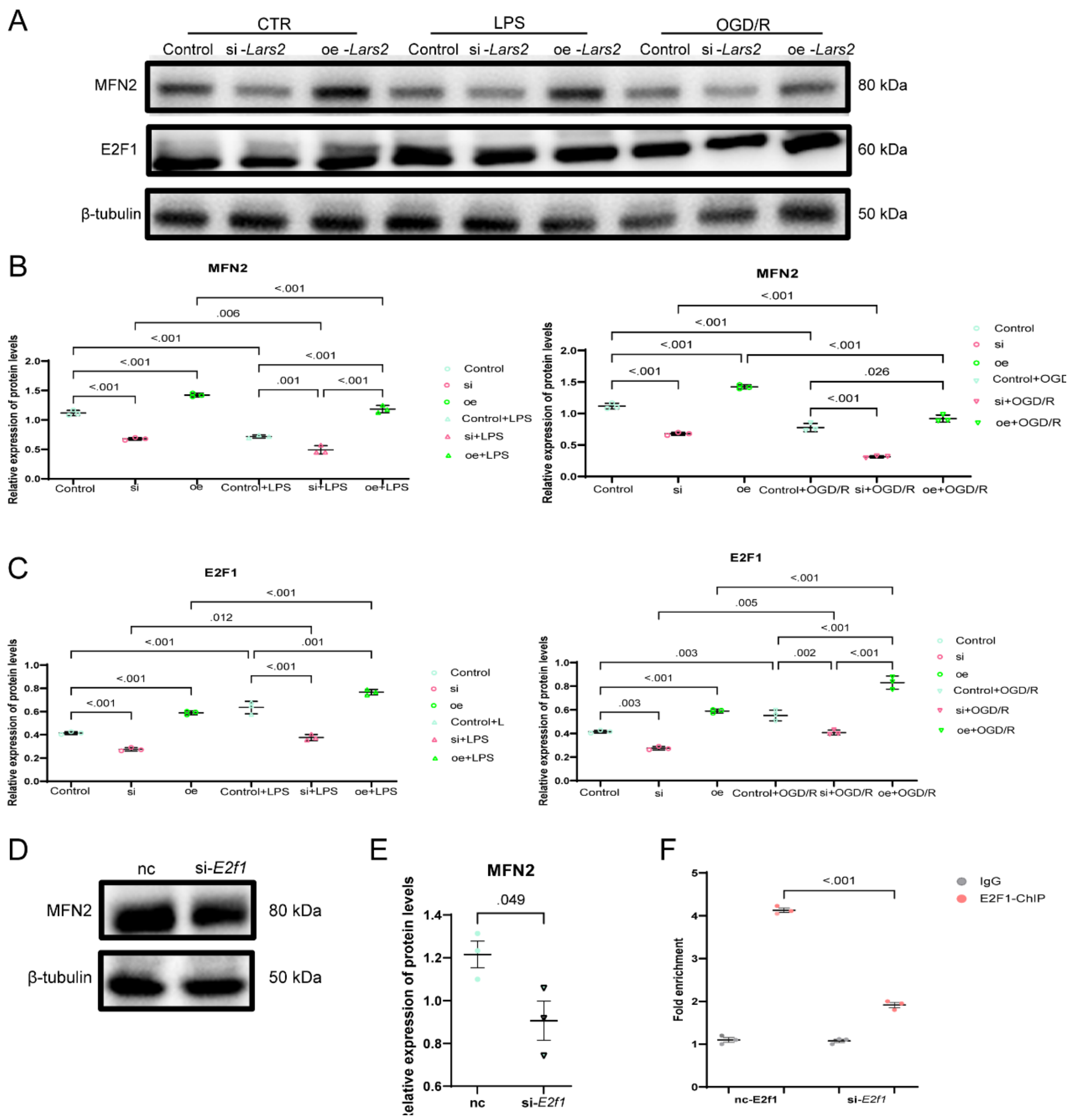

Supplementary Figure 7. ***Lars2*** **regulates MFN2 expression via E2F1 in BV2 cells.** (**A-C**) Representative western blots and quantification of MFN2 and E2F1 in LPS- or OGD/R-induced BV2 cells following *Lars2* knockdown (si-*Lars2*) or overexpression (oe-*Lars2*) or respective controls. (**D** and **E**) Representative western blots and quantification of MFN2 in BV2 cells transfected with si-RNA targeting *E2f1* (si-*E2f1*) or negative control si-RNA (nc). (**F**) CHIP-qPCR analysis showing the binding of E2F1 to the *Mfn2* promoter. In (A) and (D), β-tubulin served as a loading control. For (B), (C), (E) and (F), N=3 batches of cells per group, indicating 3 biological replicates. Data normality was assessed using the Shapiro-Wilk test. The statistical significance was determined using one-way ANOVA with Tukey's *post hoc* test for (B), (C), and (F), and independent two-tailed Student's t-test for (E). P values are shown in the figures.

© 2025. Zou Q et al. Published online at http://www.aginganddisease.org/EN/10.14336/AD.2025.0387

SUPPLEMENTARY DATA

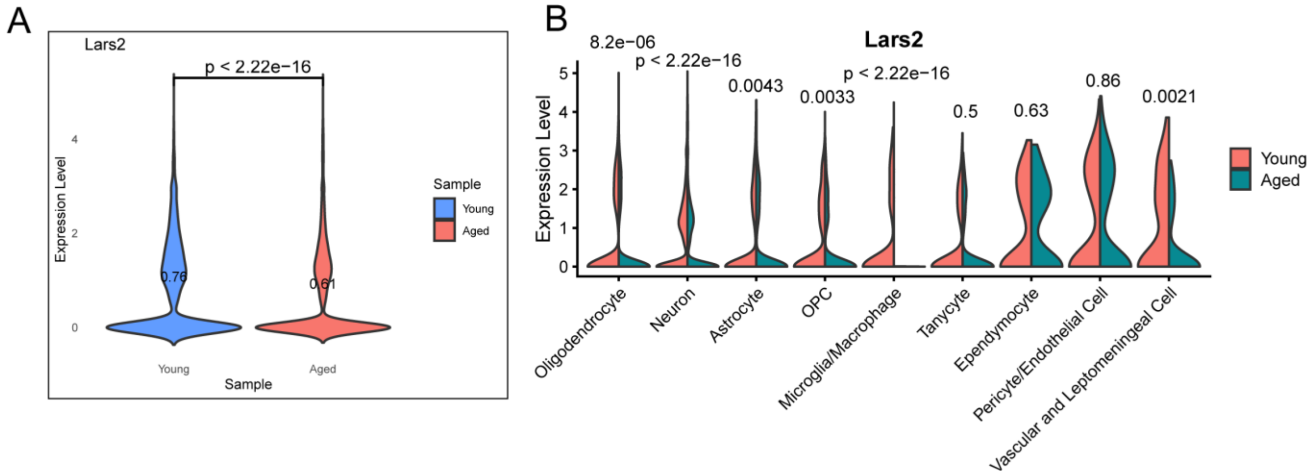

Supplementary Figure 8. Age-dependent expression of *Lars2* across different cell populations in mouse hypothalamus. (**A**) Violin plots depicting global *Lars2* expression levels in young (blue) and aged (red) mouse hypothalami. (**B**) Violin plots showing *Lars2* expression levels across different cell types in young (red) and aged (blue) mouse hypothalami.

© 2025. Zou Q et al. Published online at http://www.aginganddisease.org/EN/10.14336/AD.2025.0387

SUPPLEMENTARY DATA

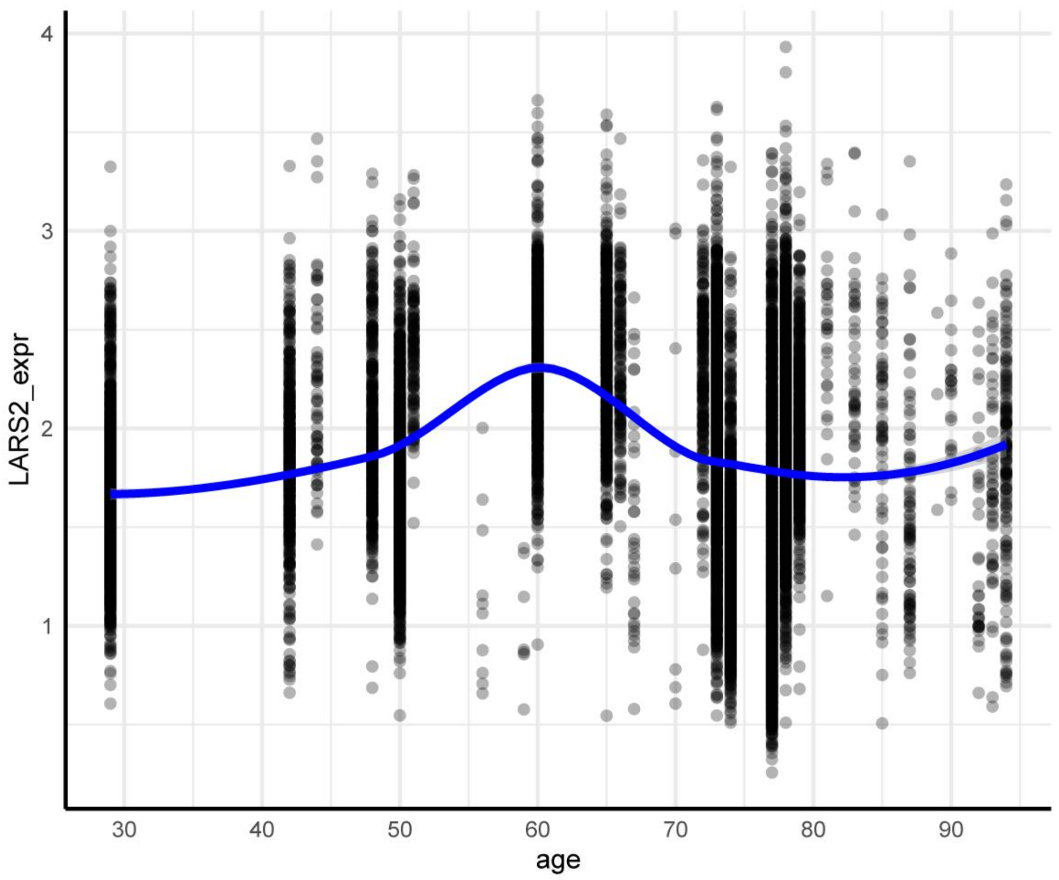

Supplementary Figure 9. Age-associated changes in LARS2 expression in human microglia. LARS2 expression in microglia peaks around age 59 and then decreases non-linearly with age, as shown by LOESS (Locally Estimated Scatterplot Smoothing) trend analysis.

© 2025. Zou Q et al. Published online at http://www.aginganddisease.org/EN/10.14336/AD.2025.0387
